# Antibody indexes in COVID-19 convalescent plasma donors: Unanswered questions

**DOI:** 10.6061/clinics/2021/e2818

**Published:** 2021-08-16

**Authors:** Tatiana Carvalho de Souza Bonetti, Flavia Roche Moreira Latini, Adriana Lukow Invitti, Marcelo Cunio Machado Fonseca, Fulvio Alexandre Scorza, Maira Garcia Saldanha, Fernanda T. Bellucco, Natalia B.S. Bacarov, Michel M. Soane, Manoel João Batista Castelo Girão

**Affiliations:** I Laboratorio de Ginecologia Molecular, Departamento de Ginecologia, Escola Paulista de Medicina/Universidade Federal de Sao Paulo (EPM/UNIFESP), Sao Paulo, SP, BR.; II Centro de Neurociencias e Saude da Mulher “Professor Geraldo Rodrigues de Lima”, Departamento de Ginecologia, Escola Paulista de Medicina/Universidade Federal de Sao Paulo (EPM/UNIFESP), Sao Paulo, SP, BR.; III Associacao Beneficente de Coleta de Sangue (COLSAN), Sao Paulo, SP, BR.; IV Disciplina de Neurociencia, Departamento de Neurologia/Neurocirurgia, Escola Paulista de Medicina/Universidade Federal de Sao Paulo (EPM/UNIFESP), Sao Paulo, SP, BR.; V EUROIMMUN Brasil, Sao Caetano do Sul, SP, BR.

**Keywords:** SARS-CoV-2, COVID-19, Antibodies, Disease Severity

## Abstract

**OBJECTIVE:**

Severe acute respiratory syndrome coronavirus 2 (SARS-CoV-2) infection is characterized by high contagiousness, as well as variable clinical manifestations and immune responses. The antibody response to SARS-CoV-2 is directly related to viral clearance and the antibodies' ability to neutralize the virus and confer long-term immunity. Nevertheless, the response can also be associated with disease severity and evolution. This study correlated the clinical characteristics of convalescent COVID-19 patients with immunoglobulin A (IgA) and IgG anti-SARS-CoV-2 antibodies.

**METHODS:**

This study included 51 COVID-19 health care professionals who were candidates for convalescent plasma donation from April to June 2020. The subjects had symptomatic COVID-19 with a polymerase chain reaction-confirmed diagnosis. We measured anti-SARS-CoV-2 IgA and IgG antibodies after symptom recovery, and the subjects were classified as having mild, moderate, or severe symptoms.

**RESULTS:**

Anti-SARS-CoV-2 antibodies were positive in most patients (90.2%). The antibody indexes for IgA and IgG did not differ significantly between patients presenting with mild or moderate symptoms. However, they were significantly higher in patients with severe symptoms.

**CONCLUSIONS:**

Our study showed an association between higher antibody indexes and severe COVID-19 cases, and several hypotheses regarding the association of the antibody dynamics and severity of the disease in SARS-CoV-2 infection have been raised, although many questions remain unanswered.

## INTRODUCTION

Severe acute respiratory syndrome coronavirus 2 (SARS-CoV-2), the new coronavirus, that hit Wuhan's central Chinese city in late December 2019 rapidly spread to all countries worldwide, impacting global public health. One of the main characteristics of SARS-CoV-2 is its high contagiousness. However, 85% of infected individuals present with only subclinical or mild symptoms, 10-15% have respiratory complications (e.g., severe pneumonia), and 5% require intensive care unit admission with an estimated lethality of 0.7-7%. In comparison to the previous coronavirus epidemics, SARS-CoV-2 has higher infection ability but decidedly lower mortality when compared with the 2002 Severe Acute Respiratory Syndrome (SARS) and 2012 Middle East Respiratory Syndrome (MERS), which had mortality rates of 9.5% and 34.4%, respectively (1,2). As SARS-CoV-2 has a high transmissibility rate, over 15 million people had been infected worldwide by just 6 months after the first case, imposing an increased burden on health systems worldwide. This public health scenario has fostered a global effort to study the pathophysiology of SARS-CoV-2 in an attempt to understand its transmission and susceptibility and to develop a better approach to treat the disease (www.who.int).

Studying the seroconversion of SARS-CoV-2 antibodies is of high relevance, as seroconversion is directly related to viral clearance. Following infection, several types of antibodies are produced, and they are detectable within days to weeks from the onset of symptoms, depending on the specific antibody classes (3-7). Some researchers have detected immunoglobulin M (IgM) and IgG antibodies against SARS-CoV-2 in the middle and late stages of the disease. The antibody (IgA, IgM, and IgG) test results were positive on average from day 9 after the onset of symptoms. The maximum detection of seroconversion is on day 16 for total antibodies, day 21 for IgM (100% of the seroconversions), and day 29 for IgG (97.1% of the seroconversions) (4). Others have identified that IgA can be detectable as early as on day 2 after symptom onset (8). We increased the accuracy of the tests by combining the detection of IgA with that of the other classes of antibodies (9). In parallel, another study highlighted the increased sensitivity of the diagnosis of COVID-19 by detecting IgA by enzyme-linked immunosorbent assay (ELISA), as IgA appears a little earlier than IgG (10).

Therefore, a better understanding of the serological dynamics of anti-SARS-CoV-2 antibodies is necessary to achieve more reliable diagnostic methods. It can also help identify the severity and evolution of the disease and reveal the ability of antibodies to neutralize the virus and to confer long-term immunity. Importantly, antibodies may also have direct implications for their therapeutic use (e.g., convalescent plasma), the response to vaccines, and the epidemiological evaluations to assess the population's susceptibility and to allow the creation and improvement of models of transmission. Therefore, this study correlated the clinical characteristics of convalescent COVID-19 patients with the expression of anti-SARS-Cov-2 antibodies.

## MATERIALS AND METHODS

### Patients

This study included candidates for the donation of convalescent plasma according to a previously approved protocol (National Ethics Committee [CONEP] Protocol Number 4.166.095).

We evaluated 51 health professionals from two tertiary hospitals in São Paulo, Brazil, who had symptomatic COVID-19 with a confirmed diagnosis of SARS-CoV-2 infection by polymerase chain reaction (PCR). The individuals were requested to participate in plasma donation, and those who accepted were invited to undergo blood screening for infectious diseases, as required in the Brazilian blood donation regulations, as well as testing for SARS-CoV-2 antibodies. At the time of screening, we collected peripheral blood samples to perform the routine blood donation tests following the national regulations, and IgA and IgG anti-SARS-CoV-2 antibodies were tested in the serum samples, in addition to the routine examinations.

Using a questionnaire at the time of sample collection, we determined whether the subjects had not had symptoms for at least a week. The patients’ symptoms were categorized as follows: (i) mild, mild clinical symptoms (fever, cough, fatigue, headache, myalgia, sore throat, or diarrhea) and no signs of pneumonia; (ii) moderate, respiratory symptoms and evidence of mild pneumonia; and (iii) severe, respiratory symptoms and evidence of severe pneumonia.

### Anti-SARS-CoV-2 IgA and IgG antibody detection

We performed Anti-SARS-CoV-2 IgA and IgG ELISAs using a commercial kit (EUROIMMUN) according to the manufacturer’s protocol. The optical density (OD) was measured at 450/630 nm. For both IgA and IgG, we calculated the ratio based on the OD of each sample and calibrator. According to the manufacturer's instructions, samples presenting OD ratios ≥1.1 were considered positive, between 0.8 and 1.0 borderline, and <0.8 negative.

### Statistics

Categorical variables were described as frequencies and percentages, and continuous variables as means and standard deviations. We also used independent group t-tests or analysis of variance and Fisher’s exact tests as required. For all analyses, we used SPSS 21 software (IBM Software), and *p*-values <0.05 were considered statistically significant.

## RESULTS

The 51 individuals analyzed showed symptoms of COVID-19, with SARS-CoV-2 infection confirmed by reverse transcription-PCR, and recovered from the infection. Their ages ranged from 24 to 68 years, and 16 (31.4%) were women, while 35 (68.6%) were men. Twenty-seven patients had mild symptoms (52.9%), nine moderate symptoms (17.7%), and 15 severe symptoms (29.4%). The mean period between symptom recovery and blood sample collection was 24.9±9.7 days, and the characteristics of the subgroups are shown in Table 1. The subgroup of severe symptoms was statistically different from the other two, while the subgroups of mild and moderate symptoms were similar in all the parameters evaluated.

Both anti-SARS-CoV-2 IgA and IgG antibodies were positive in most patients (n=46, 90.2%). Two patients were IgA- and IgG-negative, four were IgA- and IgG-positive, and two were IgA-positive and IgG-negative. We excluded all patients with any negative antibodies from the subsequent analyses.

**Figure 1:**
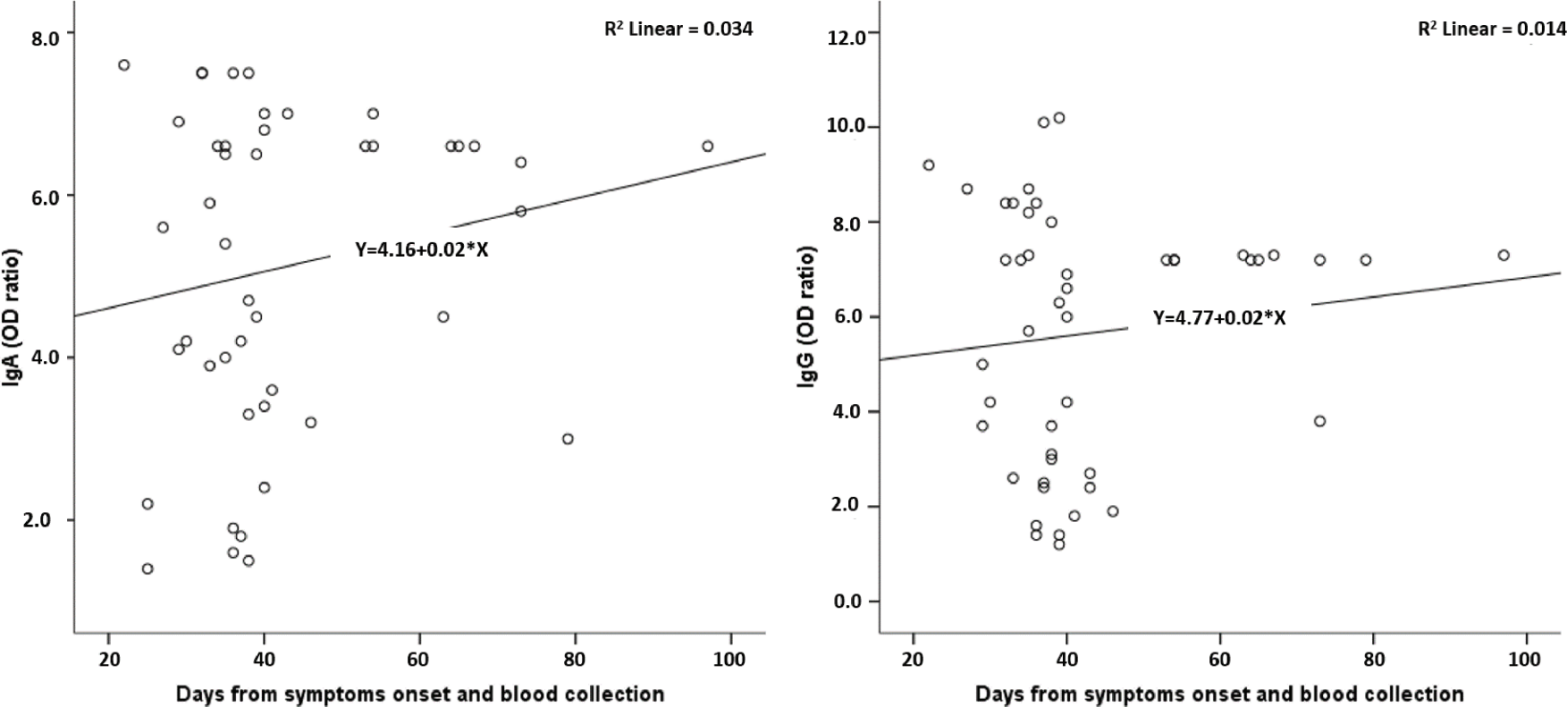
Correlation of IgA and IgG OD ratio with days from symptoms onset to blood collection.

Figure 1 shows the test reactivity variation for IgA and IgG anti-SARS-CoV-2 (OD ratios) according to the time between the onset of symptoms and blood collection. The period between symptom onset and blood collection varied from 22 to 97 days. There was no significant variation in the reactivity for both IgA and IgG.

**Figure 2:**
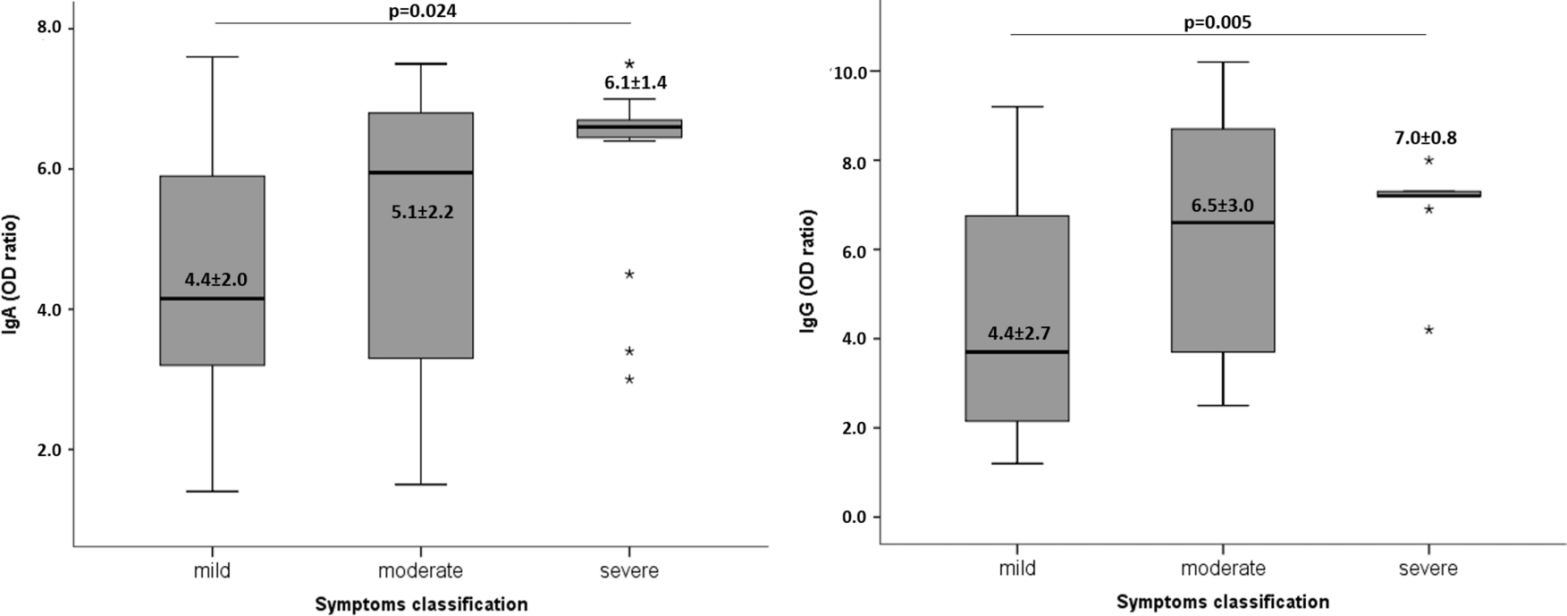
Boxplots representing the IgA and IgG OD ratios according to symptom classifications. The box values are means±standard deviations, and analysis of variance performed comparisons with Bonferroni as an ad-hoc test. The significant *p*-values were included in the figure.

We evaluated the test reactivity for IgA and IgG anti-SARS-CoV-2 (OD ratios) according to the symptom classification (Figure 2). IgA and IgG antibodies were not significantly different between the patients presenting with mild or moderate symptoms, although they were significantly higher for those with severe symptoms.

## DISCUSSION

The COVID-19 pandemic is still growing in many countries, and there is currently no effective treatment available (www.who.int). Hence, epidemiological assays to evaluate the burden of SARS-CoV-2 infections, vaccine trial outcomes, and development of therapeutics based on antibodies are urgently needed. Therefore, understanding the antibody dynamics in response to SARS-CoV-2 infection and neutralizing capacity of these antibodies is essential. Our data showed that most infected patients develop IgA and IgG anti-SARS-CoV-2 antibodies, and the antibody levels are maintained for at least 50 days after recovery from symptoms. Moreover, the reactivity of the test, which represents the antibody titers, was higher in patients with severe symptoms. These outcomes corroborate those of other studies demonstrating an association between disease severity and antibody responses (6,9-11). An important observation from our results is that IgA and IgG indexes are homogeneous in patients with severe symptoms. In contrast, patients with mild and moderate symptoms had high variability in antibody indexes.

Although the virus incites antibodies against all viral proteins by the immune system, neutralizing antibodies are crucial for immunological protection against SARS-CoV-2 (12). SARS-CoV-2 has four essential structural proteins: spike (S), nucleocapsid (N), membrane (M), and envelope (E) proteins. We performed an assay targeting the S protein, which is supposed to be the dominant protein for the antibody response. It is responsible for blocking the entry of the virus through the angiotensin converting enzyme 2 receptor, preventing viral infection of epithelial cells. Sun et al. (13) showed that IgM and IgG response antibodies against the N and S proteins of SARS-CoV-2 increased with the disease course in patients who did not need intensive care. Despite the two classes of antibodies beginning to be positive simultaneously, there was an IgM to IgG class switch in most patients around the third week of the onset of symptoms and an inverse correlation between IgG antibodies against S protein and non-specific markers of inflammation.

On the other hand, the antibody kinetics in patients who required intensive care were chaotic. Patients with severe disease tended to produce more IgM and IgG antibodies against the N protein than the S protein. In addition, they had slower IgM to IgG class-switch, suggesting that antibodies against S proteins and early class-switching of IgM to IgG may help predict a better outcome of COVID-19 (13). Furthermore, the IgA response against the S protein appears to show high and persistent levels. It is more related to early detection than the IgM response (14), and it is positively associated with the severity of COVID-19 (9). Moreover, the neutralization antibody response, mainly IgG, is primarily directed against the spike protein with higher sensitivity and earlier response to the S antigen *versus* the N antigen (15). In this context, it is crucial to evaluate the differences in the SARS-CoV-2 antibody detection methods because the antigens used to identify antibodies and follow-up times can affect the interpretation of the outcomes.

In our study, we evaluated the IgA and IgG antibodies against the S protein using a commercial ELISA kit from EUROIMMUN. A previous study using a plaque reduction neutralization test (PRNT), a reference test for serologic analysis, tested the performance of different ELISAs to detect antibodies among PCR-confirmed COVID-19 patients. The study showed a high correlation (Spearman σ value=0.93) between PRNT_90_ (90% plaque reduction neutralization test) and IgA EUROIMMUN ELISA and IgG EUROIMMUN ELISA (Spearman σ value=0.88) (5), both used in this study. Another study from the same research group, including a higher number of samples, confirmed that the S1 based IgA ELISA by EUROIMMUN had a good sensitivity to support clinical diagnosis in hospitalized patients. It also showed the best quantitative relationship with neutralizing antibodies, particularly once neutralizing titers were higher than 80 in the PRNT_50_ (50% plaque reduction neutralization test). Based on their data, it is also possible to observe that when the IgG OD ratio is 5.0, the neutralizing antibody titer in the PRNT_50_ is at least 640 (16). The recovered patients in our study who had severe symptoms had a mean OD ratio for IgG antibodies of 7.0, which indicates that the titers of the neutralizing antibodies are also high.

The relationship between viral loads and antibody titers has been previously described (17,18). However, only a few studies have addressed the kinetics of antibody responses, suggesting that antibody response is associated with disease prognosis. Herein, we showed that higher antibody indexes appear to be directly related to disease severity. The severe symptoms group had a higher index of IgG and IgA, and both were very similar. Yu et al. (8) discussed the hypothesis that enhanced IgA response in severe cases might confer damaging effects in severe COVID-19 and can be, at least in part, an IgA-mediated disease, related to IgA deposition and vasculitis.

However, cytokine release syndrome (CRS) appears to be one of the major causes of disease severity (19,20), similar to SARS and MERS (21). CRS is also commonly associated with antibody immunotherapies, bispecific antibodies, and adoptive T-cell therapies (22). Therefore, it is interesting to determine whether the increased antibody titers in severe COVID-19 disease are just a consequence of higher viral load or one of the factors responsible for the occurrence of CRS, increasing COVID-19 severity. Another hypothesis in the literature associates neutralizing IgG antibodies and the severity of the disease. This association suggests that while neutralizing IgG antibodies can prevent epithelial cell virus infection, they might also be mediators of antibody-dependent infections of leukocytes and play a central role in dysfunctional cellular responses. This mediation suggests that high antibody titers might be more associated with disease severity than with immunological efficacy. Therefore, mild, moderate, or asymptomatic patients may have low or even no neutralizing antibodies (23).

Similarly, a study evaluating SARS demonstrated the presence of IgG anti-spike proteins before viral clearance skewed the macrophage response and interleukin-8 production. The authors observed that the deceased patients displayed pulmonary pro-inflammatory monocyte/macrophage accumulation and faster neutralizing antibody responses (24). More recently, based on reinfection cases whose evolution was worse than the first SARS-CoV-2 infection, there has been a discussion on the possibility that antibodies produced in response to the virus could help, rather than fight, the virus during a reinfection (25). This phenomenon, called antibody-dependent enhancement, was found in SARS and MERS and is of utmost importance for vaccine development (26-28).

## CONCLUSION

Our study showed an association between higher IgA and IgG antibody indexes in severe COVID-19 cases, raising several hypotheses regarding the association of antibody dynamics and the severity of the disease in SARS-CoV-2 infection. Despite the limitations of this study and impossibility of evidence of a cause-effect relationship between antibody levels and the severity of COVID, we raise many questions. Why are the high levels of neutralizing antibodies found in severe SARS CoV-2 cases not enough to control the disease? Are the high indices of antibodies in severe cases a trigger for CRS? If so, why do some patients with high levels of antibodies show only mild or moderate disease? Is there a balance between triggering CRS and the neutralizing action? If so, what is this balance?

## AUTHOR CONTRIBUTIONS

Bonetti TCS was responsible for designing research studies, conducting experiments, data acquisition and analysis, and manuscript writing. Latini FRM was responsible for designing research studies, conducting experiments, data acquisition and analysis, and manuscript review. Invitti AL was responsible for designing research studies and manuscript writing. Fonseca MCM was responsible for designing research studies, data analysis and manuscript writing. Scorza FA was responsible for designing research studies and manuscript review. Saldanha MG was responsible for conducting experiments, and data acquisition. Bellucco FT was responsible for providing reagents and manuscript review. Bacarov NBS was responsible for providing reagents and manuscript review. Soane MM was responsible for providing reagents and manuscript review. Girão MJBC was responsible for designing research studies and manuscript review.

## Figures and Tables

**Table 1:** The general characteristics of the patients included in the study split according to symptoms.

Variable	General data	Mild symptoms	Moderate symptoms	Severe symptoms	*p* (ANOVA)
Number, n	51	27	9	15	
Patients age (years), Mean±SD	41.2±13.4	35.5±10.1^a^	38.4±9.0^b^	53.1±13.7^a,b^	<0.001
Time of symptoms (days), Mean±SD	18.2±9.6	14.6±7.6^c^	15.0±4.8^d^	24.5±10.0^c,d^	<0.001
Time between onset of symptoms and blood sample collection (days), Mean±SD	43.1±15.2	37.1±10.3^e^	37.8±2.6^f^	56.9±18.3^e,f^	<0.001

Mann-Whitney: a. *p*<0.001; b. *p*=0.018; c. *p*<0.001; d. *p*=0.004; e. *p*<0.001; f. *p*=0.007.
